# Medical Science and the Anti-Vivisectionists

**Published:** 1897-06-26

**Authors:** 


### Editor's Letter-Box.

[Our Correspondents are reminded that prolixity is a great bar to publication, and that brevity of style and conciseness of statement greatly facilitate early insertion.]

### MEDICAL SCIENCE AND THE ANTI-VIVISECTIONISTS.

Mr. Edward Berdoe, L.R.C.P.Ed., M.R.C.S.Eng., writes: By no law of logic or common sense would an anti-vivisectionist surgeon be compelled to surrender any atom of real knowledge once dowered to the world. No doubt many savages were poisoned by using the medicinal herbs from which many of our most valuable drugs are derived. Surely you would not say I must not give my patients belladonna or hyoscyanus because primitive man often perished by the ignorant use of them. I do not think my anti-vivisectionist friends would resort to the actual cautery for the arrest of bleeding, nor allow aneurisms to burst or operation wounds to become foul because experiments on living animals are supposed by some people to have taught us the right method of dealing with these matters. Both Celsus and Galen described the double ligation of arteries. Archigenes wrote on the tying of vessels for the arrest of hæmorrhage, and a pair of forceps for taking up an artery was dug up from the ruins of Pompeii. Rufus of Ephesus practised the torsion of arteries for stopping bleeding. Antyllus (circ. A.D. 300) tied the artery for aneurism above and below the sac and evacuated its contents. Hippocrates dressed his amputation wounds with tar water, and so was the father of the carbolic treatment if not of antisepticism; but surely it hardly needed a Lister to tell us surgeons to be a wee bit cleaner than we were in the dirty old days, when the mattress of the operating theatre was foul with the dried blood and pus of years of use. When you have discovered the cure for cancer by experiments on animals it will be time enough to tell anti-vivisectionists what is their correct attitude towards it, but you have no more monopoly of the use of the ligature for bleeding and aneurism, and of the cleanliness of the dairymaid elevated into a surgical ritual, than we.

*** We must confess to some surprise at finding an anti-vivisectionist appeal to logic and common sense. Even if Mr. Berdoe's statements about ancient practice were true, all they would prove is that right methods, such as torsion or antiseptic dressing, fall stillborn to the ground unless they rest on a basis of scientific experiment and established principle; but, as a matter of fact, arteries were not known to contain blood at the time referred to, and it is, therefore, not clear why they should have been twisted to arrest bleeding. If the staff of the proposed hospital are to use the knowledge obtained by others by means of experiments on animals, and are only to be debarred from obtaining new knowledge for themselves, they will, of course, only be a year or two behindhand in their methods of treatment. Things might be worse. But what about the honesty of the outcry against experiments on animals if the anti-vivisectionists maintain their right, as Mr. Berdoe here does, to use for their own benefit all knowledge "once dowered to the world" whether by vivisection or otherwise? We see the anti-vivisectionists in their true colours when we find them admitting that they would use for their own benefit knowledge "once dowered to the world," even though they might believe that it was gained by the "cutting up of animals alive," while they all the time hold up their hands in horror at the idea of vaccinating a guinea-pig to save a human life.—Ed. *T.H.*]

